# Animal welfare deserts: human and nonhuman animal inequities

**DOI:** 10.3389/fvets.2023.1189211

**Published:** 2023-07-11

**Authors:** Laura Reese, Xiaomeng Li

**Affiliations:** ^1^School of Planning, Design and Construction, Michigan State University, East Lansing, MI, United States; ^2^School of Global Integrative Studies, University of Nebraska-Lincoln, Lincoln, NE, United States

**Keywords:** one health, animal welfare deserts, animal welfare, spatial inequities, pet resources in distressed areas

## Abstract

Residents of distressed areas of inner cities have less access to many of life’s necessities and amenities than their more well-off counterparts. Geographic proximity has been identified as a primary barrier to accessing care for pets potentially creating animal welfare deserts. This project addresses three questions: Are there visible animal welfare deserts in distressed urban centers?; What human inequities are most strongly related to animal welfare deserts?; and What might be done to address these inequities? Using business location and census data in the city of Detroit, this research identifies distinct animal welfare deserts finding that more prosperous areas have more pet support resources and that the need for services is not related to the location of pet stores and veterinary offices. The study concludes that the overlap between human economic distress and pet resource deserts presents a threat to the goals of One Health. Potential policy solutions are proposed to address inequities in the distribution of animal welfare resources.

## Introduction

Access to veterinary care and other pet supportive services (e.g., grooming, behavior training, pet supplies) has been increasingly recognized within the animal welfare sector as a substantial barrier to the health and welfare of companion animals (1).

Residents of distressed areas of inner cities have been found to have less access to many of life’s necessities and amenities than their more well-off counterparts. Scholars have identified health care and pharmacy deserts in poor and minority areas leaving low-income residents with little access to physicians, hospitals, nursing homes, and pharmacies—particularly national chain pharmacies—which are needed to access both medication and primary care (2–5). Access to healthy food options such as groceries and non-fast food restaurants has similarly been found to be limited based on race, income, and geography (6–8). Residential segregation leads to less access to primary care physicians in majority African American zip codes creating socioeconomic and geographic barriers to accessing health care (9).

Pet ownership within urban areas has been growing around the world (10) making companion animals part of the urban ecosystem (11). Pet ownership also takes place at all income levels (12, 13) and pets have been found to increase owner well-being to the extent that caretakers are willing to pay their attendant costs (11). But it has been noted that there is “a yawning divide between rich and poor pet owners that shows no signs of abating” (Khuly, 2017 cited in (14), p. 1,121). Lack of geographic proximity has been identified as a primary barrier to accessing care for pets (15, 16) due to distance decay, i.e., when costs and distance increase, willingness to access services decreases (17), potentially creating animal welfare deserts. Thus, deprivation increases both human and nonhuman animal mortality (18). The concept of One Health emphasizes that human and nonhuman animal health are intricately intertwined. The potential for both human and pet resource deserts threatens the health and welfare of both.

Using business location and census data from the City of Detroit, this research explores these issues by addressing the following questions:

- Are there visible animal welfare deserts in distressed urban centers?
- What human inequities are most strongly related to animal welfare deserts?
- What might be done to address these inequities?

## Literature review

The literature review presents research on a chain of factors that serve to endanger both human and non-human animal welfare in distressed areas. First, inner city residents are often challenged in accessing necessities to support their own health and welfare. The first section of the literature review discusses findings related to limited access to human health resources in distressed areas. Second, because companion animals also reside in destressed areas and are treasured by their families, the same limited access to pet services can endanger their welfare and place stress on the families that love them. Extant studies have suggested that access to veterinary care and healthy and affordable pet food options is particularly problematic. Pets also provide benefits to their families and the larger community which can be limited due to lack of support services. Research documenting the presence and importance of pets in urban areas and lack of access to veterinary and pet food resources is discussed in the second section. Finally, studies have identified a number of relationships between demographic variables and animal welfare which are discussed in the third section of the literature review.

### Access to human welfare necessities in distressed areas: health care, food, products, and services

Most people are aware of how poverty and structural inequality create challenges and barriers to accessing healthy food, education, jobs, health care, and housing. There is less awareness of how limited affordable veterinary and pet wellness services create similar obstacles [(19), p. 40].

Insufficient access to necessities has been noted as a problem in economically distressed areas. This lack of access in urban areas is the result of several confounding factors: the dedensification of many urban neighborhoods resulting from the exodus of residents and businesses that meet personal needs; the fact that lower income individuals can be limited in the distances they can travel to goods and services due to transportation barriers; and because of income constraints (4, 20, 21). The neighborhood built environment and socio-economic disadvantage limit access to health care, food, and other necessities and amenities in many distressed areas (4). While needed services may be available in nearby communities, because of low access to transportation, they may not be geographically accessible (22). Based on the social determinants of health framework, access to services is not simply geographic but is also related to social and community factors such as economic status and stability, education options and quality, and the built environment (23).

While inner city residents appear to be willing to travel longer distances if products and prices were judged to be better (24), the purpose of a trip also matters in assessing distance to amenities and wiliness to travel (4). People have been found to travel the longest distances for work or school followed by recreation, personal business, and finally, shopping (25). It is unclear, however, how far pet owners are willing and able to travel to access supplies, food, and veterinary care.

While distressed areas have been found to lack certain necessities such as healthy food and medical care, gentrifying neighborhoods have accrued many amenities that cater to higher income residents. For example, such areas have been found not only to have better access to medical care but also good transportation connectivity and natural amenities such as greenspace and water access (26). Arts organizations, particularly those that are larger with robust budgets, appear to locate in neighborhoods with higher population density, young singles, and many urban amenities (27–29). Research in this vein has identified young, educated, higher income, and technology savvy residents, as the “creative class,” arguing that catering to them in terms of a host of urban amenities will lead to economic growth city-wide (30–32). For many cities, the presence of the creative class is important in understanding the economic health of different areas as well as the location of a variety of amenities ranging from coffee shops to dog parks (33, 34). Gentrifying neighborhoods may also have younger residents, without children, but with the resources to spend on non-human family members (35–37). It is thus possible that animal resources such as pet stores, veterinarians, grooming salons, and daycares are just another manifestation of businesses following younger and higher income households in their locational choices.

### Access to animal welfare necessities: food, supplies, and veterinary care

It is estimated that 23 million pets live in homes and communities which are underserved in terms of access to routine and preventative veterinary care (38).

Access to pet support items and services is important for a number of reasons. Companion animals bring many benefits to their owners as well as the larger community. They can serve as “social lubricants” facilitating interactions between humans (39). For children living in distressed communities pets can represent an exposure to nature that can reduce delinquency and crime (40). Because of the contribution of pets to social capital and human health it has been suggested that they can also be good for the local business climate (41, 42). Dog ownership specifically is related to better physical and mental well-being and sociability (6, 43, 44). Interactions with other pet owners appear particularly important for older adults because animals increase feelings of trust and encourage owners to venture out into the city (45, 46).

Increasing numbers of dogs in urban homes have raised the demand for, and number of, urban dog parks which provide benefits to both the dogs and humans: physical activity, socializing with neighbors, enjoyment of the outdoors, and increased feelings of personal safety in the park (47–50). Seeing other individuals walking dogs also increases perceptions that a particular neighborhood is safe (6).

There are demographic and spatial issues which potentially limit the community benefits of companion animals, however. Research has indicated that there is either no correlation between household income and attachment to pets or that lower income owners are actually more attached to their animals (13, 51). Indeed, it has been found that pets are common among homeless people and provide support and affection decreasing feelings of loneliness and depression. At the same time, owning a pet can reduce utilization of homeless shelters, other forms of housing, job search support, and access to medical doctors among the homeless because of limitations on services for their pets (52, 53). Pet ownership can be a barrier to securing rental housing as many apartments and other rentals bar animals or particular types of animals such as bully breeds (54, 55). Keeping an animal in violation of rental agreements can lead to eviction (55). Given the increase in households renting in the US this may be particularly problematic leading to housing insecurity and pet relinquishment to animal shelters (55–57).

Veterinary care is a critical support service for companion animals both to ensure their health but also to keep them in their homes. While findings have been somewhat mixed regarding reasons for relinquishment to animal shelters—dog-related such as behavior issues versus people-related such as moving, allergies, housing insecurity, or cost—many studies are now suggesting that changes in owner circumstances and household stress are more important in relinquishment than anything having to do with the animal (58–60). Limited access to veterinary care has been pointed to as a reason for the relinquishment of owned pets to animal shelters (59). The frequency of veterinary care, in particular, is related to family income (61). However, pet owners in low-income communities identified not only affordability of pet care services as being important but also geographic proximity, language access, and attaining information about pet supportive services and programs in the area (1, 62). Dog owners of color and those with lower household incomes were most likely to point to transportation as a barrier in accessing veterinary care for their pets (16). However, when structural barriers to pet care such as cost and proximity are removed, race appears to have no impact on efforts to seek support for pets (16, 63, 64).

In addition to lack of access to veterinary care, pet food insecurity is also problematic in urban areas (65) and varies geographically (66). Research on human food insecurity has repeatedly noted the correlation between neighborhood poverty and lack of access to grocery stores and an increased reliance on convenience stores (67). This spills over to access to pet food since, while many convenience stores may sell pet food, prices may be higher and variety and quality lower (66). Many human food banks do not provide pet food or do not have the quantity, types, and quality needed (66). In areas where car ownership is low and public transportation spotty, accessing animal care services is even more difficult, particularly since most public transit options do not allow animals (19, 38). Further, lack of a car makes shopping for pet food in bulk from discount stores or accessing pet food banks challenging because it is burdensome to try to take large bags of food on public transportation or to walk any distance with them (12, 19, 66, 68).

### Demographics of residents, human fiscal distress, neighborhood attributes, and animal welfare

Considering an increase in pet owners, the interests and needs of this distinct stakeholder group need to be taken into account in urban planning and management [(11), p. 1)].

Research examining a number of aspects of animal welfare has found correlations between community demographics and dog bites, animal cruelty, pet ownership, and attitudes toward feral cats and other wildlife (69–71). Human fiscal distress has led to stray and feral dog populations in some cities (72). Risks for humans and animals are not evenly distributed across neighborhoods. The negative health consequences of dog bites fall more heavily on persons of color; death rates from bites can be twice as high for black persons than white persons, and Hispanics and Native Americans are more likely to be hospitalized due to the seriousness of their injuries (73, 74). Injury prevalence is higher in low-income neighborhoods with greater racial and ethnic diversity as the result of the interactions between the built environment (empty lots, vacant housing) and poor economic conditions (75, 76). Many of these findings are in line with social determinants of health theories that relate the environment to health risks for individuals (77).

In other research, spatial analysis has been used to identify correlations between neighborhoods with high levels of animal cruelty, domestic violence, child abuse, crime, Hispanic populations, and abandoned properties (78). In a suburban setting, block groups with higher levels of social stress (female-headed households, percent below the poverty level, unemployment, economic disadvantage, ethnic heterogeneity, housing tenure instability, and divorced and separated persons) were found to have more reported animal cruelty offenses (79). General community hardship such as crowded housing, poverty, low incomes, percent of residents without a high school diploma, crime, and dependent children and seniors also appears related to animal crimes (80).

Community traits such as the demographic characteristics of the residents, urban or rural nature of the area, and economic conditions have also been tied to the number of residents that have pets which can have implications for animal services. For example, dog ownership was found to be higher in rural communities (81). Other demographic variables such as race, socio-economic status, and income have been found to be related to pet ownership with higher income and white residents more likely to have pets (82, 83). Finally, the nature of the local community has been found to be related to the type of animal shelter or rescue it is served by, how dogs arrive at the shelter, and to shelter outcomes. Areas with greater economic stress and lower educational attainment are more likely to have a municipal shelter, which increases stray intake, and ultimately, euthanasia (84). Thus, research has documented connections between human hardships and greater need for animal welfare services.

### Hypotheses

While there has been notice of potential pet resource deserts there are some gaps in this body of research. Generally little work has empirically examined veterinary deserts in distressed communities, with more recent research focused outside the US (15). Many studies have used survey methodologies to explore resource availability in low-income areas focusing on those working in animal welfare organizations (59), residents in low-income communities (1, 38, 66), or veterinary medical students (62). More limited research has used mapping to identify resources such as pet food pantries and veterinary offices (1, 12); recent studies on the location of veterinary clinics identified the number of veterinary clinic employees per 1,000 pets estimated at the county level (85). The previous research just discussed suggests a number of variables that might be related to the spatial accessibility of pet support resources and the need for such services: economic distress, race, housing insecurity, and market forces such as gentrification that come with the creative class. Based on the forgoing literature review the following hypotheses are tested:

*H1*: Pet resources will be located in areas with higher residential economic health.

*H2*: Pet resources will be located in areas with greater gentrification in the form of creative class residents.

*H3*: Pet support resources will be located in areas with greater need for services.

The first two hypotheses are compatible in that they tell an economic story where businesses such as pet stores and veterinarians will be located near residents that will be able to pay for services. The third hypothesis is in opposition to these suggesting that pet support resources could be located in areas with greater need for such resources due to the types of animal welfare concerns—bites, animal cruelty—noted as being more prevalent in distressed areas.

## Materials and methods

### The case

This research focuses on access to animal welfare services in the City of Detroit. Animal welfare issues in Detroit are exacerbated by several interconnected factors: economic distress, housing vacancy and abandonment, number of stray and feral dogs, high risk of dog bites, and animal cruelty. Detroit experiences dog bites at rates higher than many other cities in the US (86, 87).^1^ The City also has high numbers of stray and feral dogs. While studies modeling the US dog population have assumed that the number of feral or un-owned dogs is “negligible” (88), estimates of stray and feral dogs in the City of Detroit have ranged from 3,000 to 50,000 (72).

Detroit has experienced significant levels of economic and fiscal distress which challenge the city’s ability to provide services generally and animal welfare/control services specifically. The economic decline of Detroit has now been well documented [see for example, (89–93)]. The result has been extreme economic stress for Detroit residents.

The economic misfortunes of residents have resulted in relocations leaving animals homeless and reducing already limited resources for animal healthcare, particularly for spay and neutering services. The roaming animal problem in particular, is exacerbated by foreclosures, vacancies, and structural abandonment leaving habitats for stray and feral animals to shelter and for illegal activities such as dog fighting to be conducted. Research on Detroit has shown a pattern of spatial correlation between roaming dogs, dog fighting, animal cruelty, and bite risk for neighborhoods across the city (94).

### Data

Prior studies have defined an underserved community by the lack of veterinarians and pet supply stores (1). Thus, data on the location of pet stores and veterinarians were compiled from two sources; ReferenceUSA Historic Business data (95) and Google Maps. First, lists of pet stores and veterinary offices in Detroit in 2020 were extracted from ReferenceUSA data. Then, information on each of the locations was compared and verified with the corresponding location in Google Maps; a few additional locations that existed in 2020 (verified by Google Street View) were added. In the finalized list, there were a total of 11 pet stores (4 locations added based on Google Maps) and a total of 12 vets (4 locations added based on Google Maps). This also allowed for locating these pet resources by the 27 zip codes that comprise the City of Detroit which serve as the unit of analysis for this study. Because of the low number of pet stores and veterinarians in the city, use of a smaller geography such as census tracts was not viable.

Pet support resources are limited in Detroit as a whole; as noted there are only 11 specialty pet stores and 12 veterinary offices in the city. There are four animal shelters that provide medical care to local animals to varying extents and several nonprofit organizations raise funding for low cost spay/neuter and vaccination services. These mobile clinics were not included nor were the animal shelters since their locations are the result of past land use decisions or the current availability of appropriate (cost, zoning) land.^2^ As such they do not represent decisions on the private market regarding where to locate services. It should be noted that the locations of grocery stores and chain pharmacies (that typically sell pet supplies) were also mapped but not used in the current study.^3^ On the one hand the location of these businesses tended to match the location of more specific pet resources in that zip codes with more groceries are significantly more likely to have pet stores and veterinary offices. On the other hand, because they focus on human food needs their locations may be measuring something beyond pet resource deserts more narrowly defined.

Data drawn from the 2021 American Community Survey (ACS) estimates were used to examine patterns of correlation between the location of pet resources and the demographic characteristics of the zip codes. Prior studies discussed in the literature review have included a number of variables in the context of resource deserts: economic conditions (poverty, female-headed households, unemployment, income), the presence of dependent children, education levels, lack of a vehicle, housing conditions, and race and ethnicity. These variables, drawn from the ACS are used to test H1: Pet resources will be located in areas with higher residential economic health. Two variables were added to the dataset to measure the need for animal supportive services; dog bites and animal cruelty cases per zip code. These variables are used to test H3: Pet support resources will be located in areas with greater need for services. The extant research discussed in the literature review indicates that both bites and cruelty are related to levels of human distress, most importantly economic factors (80, 94). Higher levels of bites and cruelty suggest areas that have higher needs for pet support. Data were drawn from police department reports in the category of “animal crimes” which include dog bites, animals at-large, and animal cruelty. All 302 animal cruelty incidents in Detroit between 2007 and 2015 for which there is a police report were included in the analysis. The most frequent types of animal cruelty in Detroit were shooting (23% of incidents), kicking/blunt force (21%), other (17%), neglect (12%), and dogfighting (10%). Stabbing (6%), poisoning (5%), and threatening to harm an animal (2%) are much less common. Data on dog bites were collected from police reports for the same time period with a total of 478 bite incidents for which there was a police report. Dog bite was defined as a police report of a bite—severity was neither a criteria for filing a report nor was it assessed in the report. Because of data availability, the bite and cruelty data cover a period well before the census and pet resource data. While this raises obvious concerns it does allow for a sense of whether current resources have located in areas with historically greater need for services.

### Analysis

The analysis proceeds as follows. First, frequency and visual data are used to describe the locations of pet stores and veterinarians in the city of Detroit. Second, the correlates of pet support resources *per capita* by zip code are explored. This analysis is used to test the hypotheses related to potential relationships between economic health and need for pet support services and the location of pet stores and veterinary offices. To simplify the analysis and reduce multicollinearity in the regression analyses to follow, a number of the variables were combined into three indexes based on exploratory factor analysis^4^ (Table 1). First, the residential economic health index measures median household income and percentages of residents above the poverty line, employed, not using food stamps, and that are not under 18-years old. Zip codes higher on the index have better residential economic health. Second, the number of police reports of dog bites and animal cruelty were combined into a single index representing the potential need for animal support services.

**Table 1:** Factor analysis: economic health and need for services indexes.

	Factor loading
*Economic health index*	
Median household income	0.85
% not 18 or under	0.71
% not in poverty	0.87
% employed	0.76
% not using food stamps	0.92
*Need for services index*	
#cruelty reports	0.90
# dog bite reports	0.90

A third index indicates the number of creative class residents within a zip code and was drawn from recent research on Detroit (34). Four variables were used to identify the creative class. Population concentrations of young adults aged 20 to 34 and of college graduates were used as proxy measures of the creative class. Employment in professional, scientific and technical; finance, insurance and real estate; and arts, recreation and entertainment industries represents the creative class theory’s emphasis on new economy jobs that involve talent and technology. The proportion of residents moving from a different county in the United States or from abroad provides a measure of the relative attractiveness of the location. American Community Survey data were used to calculate location quotients for these four variables (young adults, college graduates, employees in creative industries, and recent in-movers) which were summed to provide a creative class index. This index is used to test H_2_: Pet resources will be located in areas with greater gentrification in the form of creative class residents. As noted, correlation analysis was conducted between the number of pet resources and all of the demographic variables individually and as combined in the three indexes to test all three hypotheses. While the bivariate correlation analysis is instructive it does not assess which of the variables are most strongly related to pet resources and how much of the variation in the location of pet stores and veterinary offices can be explained by the variables in the analysis. To address this, regression analysis is employed with the number of pets stores and veterinarians *per capita* as the dependent variable. Independent variables include all those measures that were significantly related to the number of pet resources in the correlation analysis.

## Results

### Descriptive data

Descriptive data for the zip codes that comprise Detroit are presented in Table 2. It is clear that, despite reports of improvements in the city’s economy (89, 96, 97), many indicators still suggest a place where residents remain in distress. Average rates of poverty and female-headed households are at 45%, unemployment at 14%, and 24% of residents do not have a vehicle on average; only 14% of residents have a college degree. The average unit vacancy rate across zip codes is 25%. Standard deviations do indicate substantial differences among the zip codes on a number of these variables, particularly in population, the number of households, the percentage of white residents, median household income, and median rent. Prior research on Detroit has identified the Midtown and Downtown areas of the city as more prosperous, experiencing greater growth in population and incomes, and better able to attract young creative class individuals than other areas of the city (34, 71, 98).

**Table 2:** Descriptives on Detroit zip codes.

	Mean	Standard deviation
Population	25,665	14,234
Population density	2,506	2,092
%white persons	0.19	0.17
%under 18	0.23	0.07
%over 65	0.14	0.04
%college grad	0.19	0.13
%vacant units	0.25	0.09
Households	13,766	6,244
%owners	0.44	0.15
%female headed	0.45	0.07
%poverty	0.45	0.09
Median rent	$823	$158
%food stamps	0.36	0.09
Median household income	$32,233	$9,498
%unemployed	0.14	0.05
%no car	0.24	0.08

*N* = 27.

### Location of pet resources

**Figure 1:**
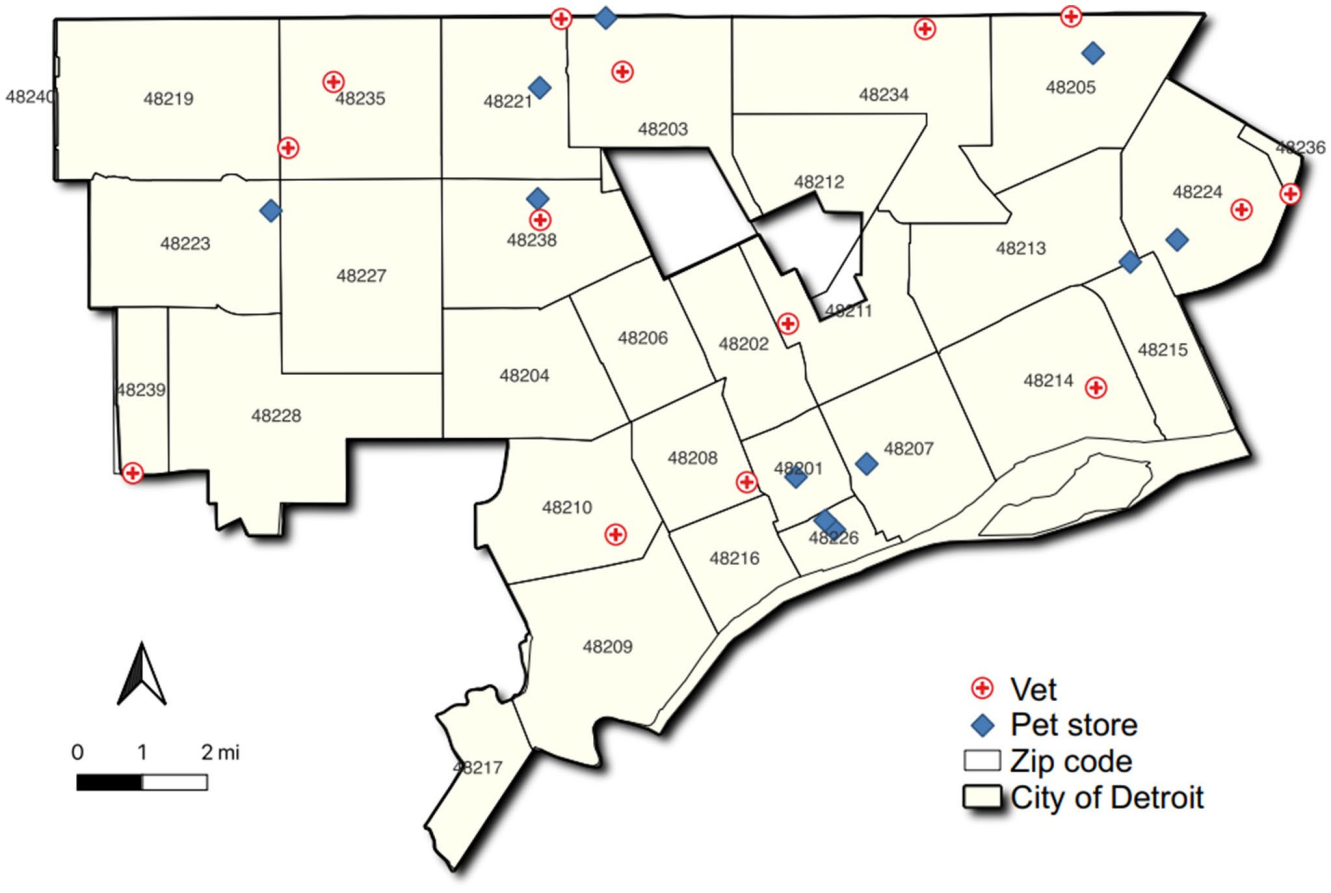
Map of the locations of pet stores and veterinarians.

A map of the location of pet stores and veterinarians is presented in Figure 1. Several trends are evident. First, as indicated by the frequency data, it is clear that the city as a whole has few dedicated pet stores and veterinarians. Second, 11 of the 26 zip codes in the city have no resources of these types at all. Third, the existing veterinarians and pet stores are not evenly distributed across the city. The zip code areas without these basic pet supportive resources are clustered in contiguous areas, further exacerbating the limited accessibility. Two large areas lacking veterinarians and pet stores are found in a band stretching across the mid-city and in southwest Detroit.

**Figure 2:**
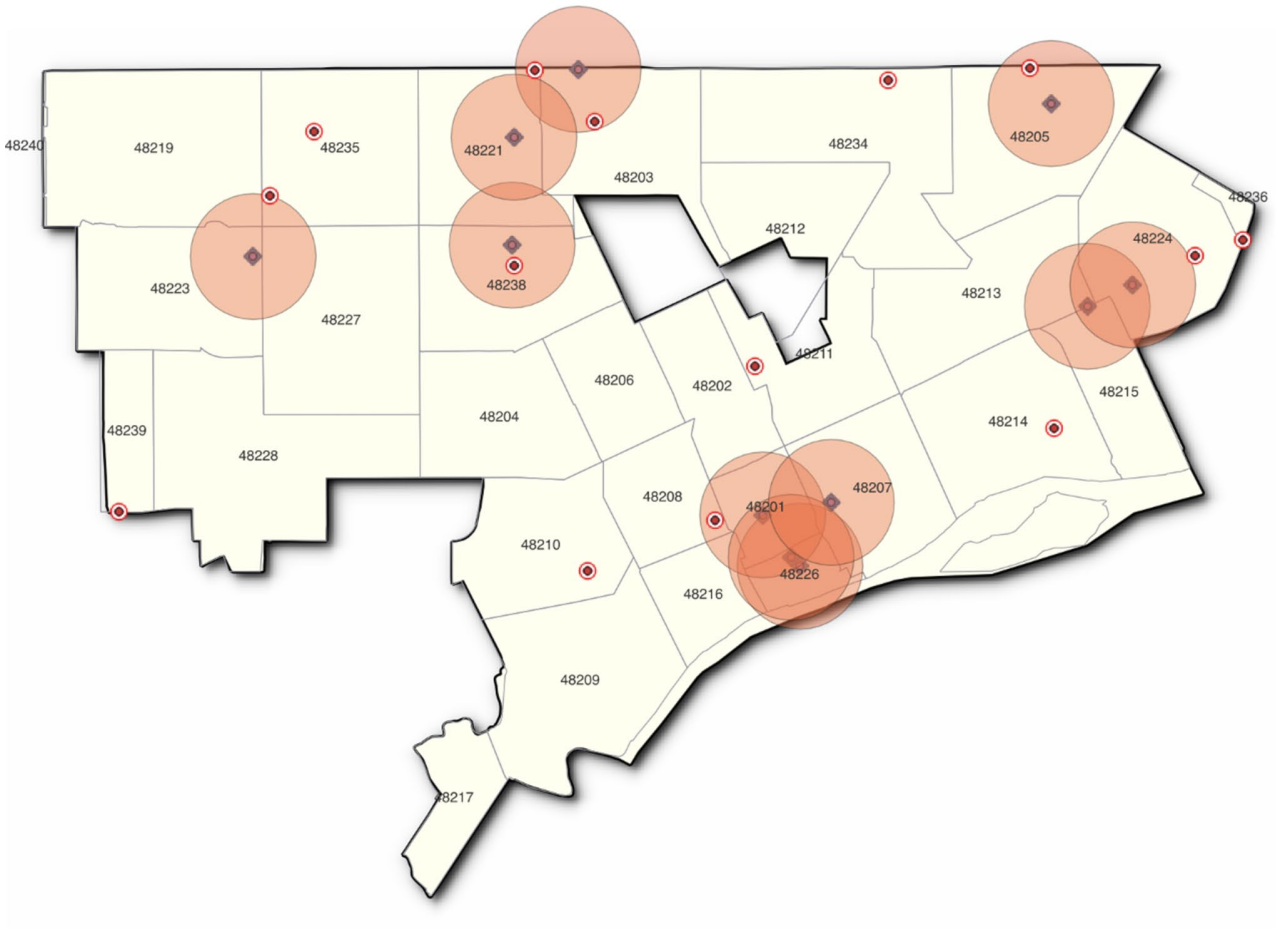
One-mile radius, pet stores.

Of the 11 specialty pet supply stores, four are in the downtown/midtown area; the others are scattered around the periphery of the city, leaving a large unserved area in between. As a result of the clustering of the pet store locations, much of Detroit is a mile or more away from a pet store (Figure 2). The neighborhoods that enjoy the best access are those with higher resident incomes or include concentrations of better-paying employment opportunities (for example, the downtown core).

**Figure 3:**
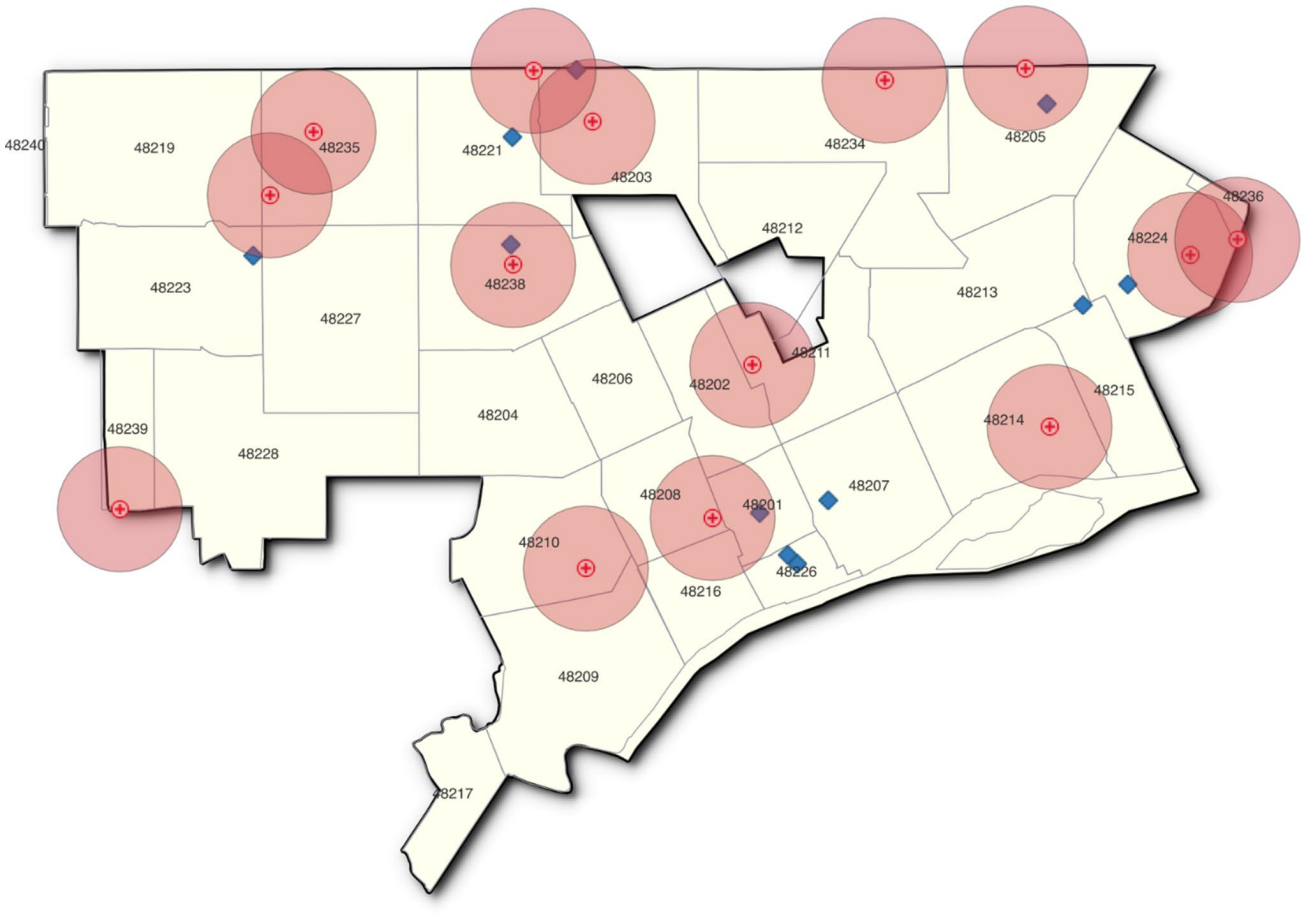
One-mile radius, veterinary locations.

The Detroit veterinary practices do not exhibit the same degree of clustering. Figure 3 illustrates that the one-mile radius around more than half of the veterinary offices does not overlap any of the other vet market areas. The veterinary offices are also more likely to be found in middle- or lower-income ZIP codes.

### Correlates of pet stores and veterinarians

Several demographic variables are significantly correlated with the number of pets stores and veterinarians *per capita* (Table 3). Zip codes with a larger percentage of college graduates, higher median rents, higher *per capita* and household incomes, and less density (lower population per square mile) have significantly more resources for pets. Zip codes with more children, residents receiving food stamps, owned homes, and housing units have significantly fewer pet stores and vets. In short, areas with greater residential economic health and more creative class residents have more pet support services. Conversely, zip codes with greater need for supportive services as indicated by higher rates of dog bites and animal cruelty have fewer pet stores and vets although the relationship does not reach statistical significance.

**Table 3:** Pearson correlations, demographics, and pet stores/veterinarians *per capita*.

Demographics	Pets and vets *per capita*
% white persons	0.25
% 18 and under	−0.47*
% 65 and older	−0.13
% college grad	0.44*
Population/square mile	−0.39*
%vacant units	−0.32
# households	−0.37
% no vehicle	0.22
% owned homes	−0.54**
% female-headed households	−0.11
% poverty	−0.16
Median rent	0.51**
% food stamps	−0.48*
*Per capita* income	0.67**
Median household income	0.47*
Unemployment rate	−0.26
Economic health index	0.44*
Creative class index	−0.61**
Need for services index	−0.31

*N* = 27; *significant at 0.05; **significant at 0.01.

### Regression analysis

The variables significantly correlated with animal resources in the bivariate analysis were entered into a regression analysis (Table 4). Because of multicollinearity, the indexes for economic health and creative class were used rather than their component variables. Together the independent variables account for 50% of the variation in pet stores and veterinarians *per capita*. Zip codes with more residents in the creative class and higher median rents have significantly more pet resources *per capita*. More densely populated areas have significantly fewer. Areas higher on the economic health index also have more pet resources although this is significant at only 0.10 in multiple regression. Creative class and median rents are the best predictors of pet stores and veterinarians *per capita* based on the beta values. A reduced regression model using just the creative class index and median rents as independent variables accounts for 40% of the variation in pet stores and veterinarians *per capita* although only the creative class index remains significant in this regression model (Table 5).

**Table 4:** Multiple regression, pet stores, and veterinarians *per capita*, full model.

	Unstandardized B	Standard error	Standardized coefficients beta	*T*	Significance	VIF
Economic health index	−2.903E-5	0.00	−0.47	−1.80	0.09	3.52
Creative class index	1.726E-5	0.00	0.59	2.53	0.02	2.77
Median rent	2.287E-7	0.00	0.59	3.05	0.01	1.92
Population per sq. mile	−9.705E-9	0.00	−0.33	−2.06	0.05	1.32
Constant	0.00	0.00		−2.08	0.05	
Adjusted *R*^2^ = 0.50						

**Table 5:** Multiple regression, pet stores, and veterinarians *per capita*, reduced model.

	Unstandardized B	Standard error	Standardized coefficients beta	*T*	Significance	VIF
Creative class index	1.393E-5	0.00	0.47	2.80	0.01	1.23
Median rent	1.186E-7	0.00	0.30	1.80	0.08	1.23
Constant	0.00	0.00		−2.18	0.04	
Adjusted *R*^2^ = 0.40						

## Discussion

Three central questions were posed in this research: Are there visible animal welfare deserts in distressed urban centers?; What human inequities are most strongly related to animal welfare deserts?; and What might be done to address these inequities? It is clear that animal welfare resources in the form of pet stores and veterinary offices are not evenly distributed and that a large swath cutting across central areas of Detroit is a pet resource desert. This supports prior work indicating gaps between rich and poor areas in access to pet care resources (14, 16).

The hypotheses tested three potential explanations; economic conditions, the location of the creative class, and need for services. The correlation analysis supported H_1_ and H_2_, specifically that pet resources are significantly more likely to be located in zip codes with more highly educated residents, with higher incomes, fewer children under 18, and higher median rent. Areas with fewer pet resources are those that are more economically distressed in terms of food stamp use and lower incomes.

The need for services, in terms of bite and cruelty incidents, was not significantly correlated with the location of pet resources; thus, H_3_ was not supported. The percentage of zip code residents without cars also did not correlate with the location of pet resources, i.e., areas with more households without personal vehicles did not have fewer resources. This finding does not conform to previous work suggesting that lack of transportation was a primary barrier to accessing veterinary care (16, 22) but may be due to the fact that Detroit has generally low levels of car ownership. And the lack of significant correlation does not mean that residents without cars have equal spatial access to pet resources, however. The map of resources indicated that many pet stores and veterinary offices were located at the borders of the city implying that distance to such locations might still be significant for a resident without a car even living in the same zip code as the resource. The very weak public transportation system in Detroit is unlikely to address such spatial inequities.

Previous research has suggested that pet ownership might constitute an additional discriminatory factor in housing; i.e., that rental housing is difficult to access with pets and perhaps, as a result, pet-friendly housing may be of lower quality (55). While the relationship did not hold up in multiple regression, the correlation analysis indicated that zip codes with higher home ownership had lower access to pet resources. This may be the result of more pets living in owned homes due to rental restrictions as well as the reality that, in Detroit, much of the higher end rental housing is located in the Mid and Downtown areas that are better served.

Multiple regression analyses lent greater support to the creative class as opposed to the economic explanation as the economic health index did not remain significantly correlated with pet stores and veterinarians *per capita*. The creative class index and median rent alone explain 40% of the variation in the location of pet resources in the city suggesting that, as with many urban amenities, location patterns in these resources tend to favor less distressed and gentrifying areas exacerbating existing inequalities (26, 33, 34).

Given this reality, what can be done to address the pet support inequities evident in Detroit? There are several ways to approach this question: via the private market, through support from the nonprofit sector, and governmental intervention. These approaches are not mutually exclusive as most efforts to affect business location involve the expenditure of government revenue. Possibilities for each of these approaches will be discussed in turn.

First, the location of pet stores and veterinary offices is market driven as with other types of businesses such as grocery stores and medical practices. Based on the strong connection between the location of the creative class and pet support resources the effects of the market are clear. From this perspective, encouraging more pet stores and veterinarians to locate in distressed and underserved areas of the city is an economic development challenge. A variety of actions, involving governmental investments, have been recommended to foster the location of smaller businesses in distressed areas which might be applied to pet resource operations. Business incubators are a well-documented means of local investment in business development (99–101). Typically, a small business incubator begins with a facility/building that provides a location for new firms, offering below market rents, and an array of support services designed to meet the needs of small, start-up firms that are often owned by inexperienced or first-time entrepreneurs. Incubators could be good locations for small specialty pet stores or new veterinary practices. Start-up capital and small revolving loan programs would support potential pet store owners in locating in distressed areas or for veterinarians to develop offices (99, 101).

The risk of a pet ending up in an animal shelter varies by community (84). Many of the reasons for relinquishment can be addressed by community programs that improve access to affordable medical care and pet supplies (58, 102). In high poverty areas, which may represent animal welfare resource deserts, education and awareness about available supports must be raised (14, 16, 19, 41, 58). Nonprofits have long provided animal welfare services and research on Detroit has suggested that they provide the bulk of pet support including health care and food provision, rescue and adoption programs, anti-tethering advocacy, and fence-building for chained dogs (98, 103). A number of animal welfare organizations around the US have been implementing programs to support those struggling financially to keep their pets providing food, low-cost medical care, training assistance, fencing, crates, and so on (HSUS, nd.). The American Society for the Prevention of Cruelty to Animals’ (ASPCA) Keeping Pets and People Together program provides animal welfare services in underserved communities including low-cost medical care, supplies, and information (19). In Detroit, nonprofit organizations such as Dog Aide and C.H.A.I.N.E.D provide a variety of pet support services including food and supplies, support for veterinary care, fencing, and education. Mobile veterinary clinics supported by nonprofits are another way to address lack of access to services in distressed areas (14). The partnership between the ASCPA and the New York Police Department (NYPD) to fight animal cruelty is an example of public and nonprofit collaboration. Here the resources of the NYPD are brought to bear on animal cruelty cases while the ASPCA focuses on the health and welfare of the nonhuman animal victims and also the needs of guardians in cases where pets may be able, with a supportive response, to remain in their homes (104).

Co-locating human and nonhuman animal food pantries or medical services represents a One Health approach to address welfare disparities. One Health programs emphasize both human and nonhuman animal welfare (1). In one sense the concept is related to the potential transmission of disease as codified in Centers for Disease Control and World Health Organization initiatives (42, 105, 106). As implemented in urban areas it can take on the form of joint facilities to provide human medical and veterinary care particularly in distressed areas. Poor and homeless people could be provided a single location to receive physical and mental health and social services for themselves and veterinary services for their pets (107). Food pantries that have both human and nonhuman animal food is another One Health approach (12). Conceivably programs combining assistance and services to both pets and their guardians could be joint ventures of animal welfare and social service nonprofits, veterinary programs, animal shelters, and community medicine organizations. Involving residents in the development of these services would help ensure that programs are stakeholder-driven and address perceptions found in other research that lower income and minoritized pet owners do not feel comfortable interacting with extant veterinary professionals (59, 62).

### Limitations and future research

This study does have some limitations that are worth noting. First, it is focused on a single city in the US; this is both an advantage and a disadvantage. While the focus on a single city allowed for a more in-depth examination of the location of pet support resources and needs and obviated the need for surveys as proxies for actual locations, it could be that findings are not generalizable to other locations and contexts. The focus on the City of Detroit does, however, allow for an analysis of pet resource deserts under conditions of severe economic stress and high levels of racial segregation. Future research should explore these issues across a variety of cities and ideally, in different national contexts.

The operation of the race variable may have been affected by the lack of racial diversity in the majority minority city, potentially accounting for the lack of correlation between race and pet resources in this analysis. Again, replicating the study in communities with greater racial and ethnic diversity would be beneficial.

The data measuring need for services—dog bites and animal cruelty—were drawn from an earlier time period than the demographic and pet resource data. A more desirable way to measure need would have been to use data from the City’s animal control agency on the location of animals that were brought in by residents or animal control officers. These data could not be accessed for the city of Detroit, however. Research on other cities that allow access to those data or have them relatively available is warranted. The small number of pet stores and veterinarians in the city did not allow for analysis to be run on smaller geographies such as census tracts or block groups. Small numbers of pet stores and veterinary offices also made it more likely to miss significant correlations; additional variables might have been correlated with pet resources with a larger sample size.

## Conclusion

This study tested three potential explanations for the location of animal welfare resources in cities: economic conditions, the location of the creative class, and the need for services. Data suggest that there are significant animal resource deserts in the City of Detroit related to economic distress for residents. Specifically, there are fewer pet stores and veterinary offices in areas with less educated residents, with lower incomes, lower median rents, more children under 18, and higher use of food stamps. More pet support services are located in areas of the city experiencing gentrification with younger and more well-off residents. The need for services, in terms of bite and cruelty incidents, was not significantly correlated with the location of pet resources.

Animal resource deserts, leaving residents of distressed areas without necessary services for their pets, constitute an important equity issue that can have far reaching implications. Inadequate access to pet supplies and veterinary care can endanger the lives of pets and increase stress on their human guardians who must worry about how to obtain food and medical care for their family members and may increase the risk of relinquishment to shelters. Human inequities can reduce access to pet care but also to affordable and high-quality rental housing, homeless shelters, and transportation services. In a larger sense, if the need for animal welfare services is not related to accessibility to pet care resources then the health and safety of both a city’s people and animals are at risk. This research demonstrates the importance of considering the One Health of human and nonhuman animal populations.

## Data availability statement

The raw data supporting the conclusions of this article will be made available by the authors, without undue reservation.

## Author contributions

All authors listed have made a substantial, direct, and intellectual contribution to the work and approved it for publication.

## Conflict of interest

The authors declare that the research was conducted in the absence of any commercial or financial relationships that could be construed as a potential conflict of interest.

## Publisher’s note

All claims expressed in this article are solely those of the authors and do not necessarily represent those of their affiliated organizations, or those of the publisher, the editors and the reviewers. Any product that may be evaluated in this article, or claim that may be made by its manufacturer, is not guaranteed or endorsed by the publisher.

## Footnotes

^1^Rates of dog bites appear to have dropped recently in cities across the nation and this also appears to be the case in Detroit based on 2016 data (https://www.scribd.com/doc/27348222/Denver-Dog-Bite-Statistics-Does-BSL-Work; http://www.detroitnews.com/story/news/local/detroit-city/2016/08/05/detroit-dog-bites-adoptions/88316656/).

^2^One exception to this is the veterinary clinic at the Humane Society of Michigan location in Detroit. This clinic regularly provides care for Detroit residents so serves as a primary veterinary resource in the city and is included in the dataset.

^3^Maps available upon request.

^4^For the factor analysis the standard SPSS defaults of varimax rotation and listwise deletion of missing data were used.
